# A variety of bacterial aetiologies in the lower respiratory tract at patients with endobronchial tuberculosis

**DOI:** 10.1371/journal.pone.0234558

**Published:** 2020-06-25

**Authors:** Sae Byol Kim, Won-Yeon Lee, Ji-Ho Lee, Seok Jeong Lee, Myoung Kyu Lee, Sang-Ha Kim, Young Uh, Soon-Hee Jung, Beomsu Shin

**Affiliations:** 1 Department of Internal Medicine, Yonsei University Wonju College of Medicine, Wonju, Republic of Korea; 2 Department of Laboratory Medicine, Yonsei University Wonju College of Medicine, Wonju, Republic of Korea; 3 Department of Pathology, Yonsei University Wonju College of Medicine, Wonju, Republic of Korea; The University of Georgia, UNITED STATES

## Abstract

Recently, our understanding of the elusive bacterial communities in the lower respiratory tract and their role in chronic lung disease has increased significantly. However, little is known about the respiratory microorganisms in patients with endobronchial tuberculosis (EBTB), which is a chronic inflammatory disease characterized by destruction of the tracheobronchial tree due to *Mycobacterium tuberculosis* (MTB) infection. We retrospectively reviewed data for histopathologically and microbiologically confirmed EBTB patients diagnosed at a tertiary referral hospital in South Korea between January 2013 and January 2019. Bacterial cultures were performed on bronchial washing from these patients at the time of EBTB diagnosis. A total of 216 patients with EBTB were included in the study. The median age was 73 years and 142 (65.7%) patients were female. Bacteria were detected in 42 (19.4%) patients. Additionally, bacterial co-infection was present in 6 (2.8%) patients. Apart from MTB, the most common microorganisms identified were *Staphylococcus aureus* (n = 14, 33.3%) followed by *Klebsiella* species (n = 12, 28.6%; 10 *Klebsiella pneumoniae*, 2 *Klebsiella oxytoca*), *Streptococcus* species (n = 5, 11.9%), *Enterobacter* species (n = 4, 9.5%), and *Pseudomonas aeruginosa* (n = 3, 7.1%). A variety of microorganisms were isolated from the bronchial washing indicating that changes in microorganism composition occur in the airways of patients with EBTB. Further studies are needed to investigate the clinical significance of this finding.

## Introduction

Endobronchial tuberculosis (EBTB) is defined as *Mycobacterium tuberculosis* (MTB) infection of the tracheobronchial tree with or without lung parenchymal involvement [1]. The reported incidence of EBTB varies from 4.1% to 54.3% [2, 3]. The pathogenesis of EBTB is not fully understood, but the beginning of infection includes direct implantation of MTB into the bronchus from the adjacent lung parenchyma or mediastinal lymph nodes, hematogenous or lymphatic spread, or implantation of organisms from infected sputum [4]. On the other hand, pathologic endobronchial mucosal changes caused by MTB range from simple erythema, submucosal congestion, caseous necrosis due to loss of mucosal integrity, ulcer formation, and fibrostenosis [4]. Although these changes are closely related to the deterioration of symptoms and prognosis, there are still limitations to a precise understanding of the variable course at EBTB [5, 6].

Chronic inflammatory lung disease harbors distinct respiratory bacterial communities and these microorganisms may be associated with the pathogenesis and prognosis of the disease [7–10]. Patients with structural lung disease such as chronic obstructive pulmonary disease (COPD), bronchiectasis, or interstitial lung disease are chronically infected with potentially pathogenic microorganisms, resulting in persistent respiratory symptoms and recurrent exacerbation of disease [11–13]. Similarly, the endobronchial mucosal changes in EBTB cause local airway inflammation, further airway damage or remodeling, and MTB can form biofilms, which can be altered respiratory microorganisms [14]. However, little is known about lung microorganisms in patients with EBTB.

In this study, we analyzed data from EBTB patients and their bronchial washing and investigated the prevalence and composition of respiratory microorganisms.

## Materials and methods

### Study population

We retrospectively reviewed data for EBTB patients diagnosed at Wonju Severance Christian Hospital, a tertiary referral hospital in South Korea, between January 2013 and January 2019. Patients were included as follows: (1) typical bronchoscopic finding of EBTB and (2) histopathologic and microbiological evidence of MTB in endobronchial biopsy specimens and bronchial washing [4]. The histopathologic findings include chronic granulomatous inflammation, chronic inflammation, erosion, ulcer, or caseous necrosis [4]. We identified 2,425 patients with active pulmonary tuberculosis between January 2013 and January 2019. We excluded 895 patients because bronchoscopy was not performed and 1,314 patients had inconsistent bronchoscopic findings or histologic features of EBTB. The final number of study participants was 216.

### Bronchoscopic procedures

All bronchoscopy procedures were performed by attending physicians at the time of EBTB diagnosis. All procedures were performed via the trans-nasal or oral route under local anesthesia. Oxygen was administered via nasal cannula and oxygen saturation was maintained at > 90% as measured by continuous pulse oximetry monitoring. Midazolam at 0.07 mg/kg was delivered intravenously when needed to achieve adequate sedation prior to the procedure. Bronchial washing and bronchoscopic biopsy were performed on suspected endobronchial lesions. Bronchial washing fluid was collected using the suction channel into a trap [15, 16]. Bronchoalveolar lavage was not performed because we did not undertake a wedge position within the selected bronchopulmonary segment due to endobronchial mucosal changes caused by MTB [17].

### Bacterial detection

All bronchial washing specimens were included in the study. All specimens were examined for bacterial pathogens using the Gram stain method and bacterial identification was performed using automatic MicroScan WalkAway 96 Plus (Beckman Coulter, Brea, CA, USA) or VITEK 2 (bioMérieux Inc., Hazelwood, MO, USA) diagnostic systems. Additionally, all specimens were stained using the Ziehl-Neelsen method and cultured using both solid (3% Ogawa medium; Korean Institute of Tuberculosis, Korea) and liquid (BACTEC 960 Mycobacterial Growth Indicator Tube; Becton Dickinson, Sparks, MD, USA) media.

Positive acid-fast bacilli (AFB) stains were described as 1+ to 4+ (1+, 1–9 AFB/100 fields; 2+, 1–9 AFB/10 fields; 3+, 1–9 AFB/field; and 4+, > 9 AFB/field) [18].

### Data collection

Demographic and clinical data were collected from medical chart review including age, sex, body mass index (BMI), smoking history, comorbidities, respiratory symptoms, chest computed tomographic (CT) findings, white blood cell (WBC), erythrocyte sedimentation rate (ESR), C-reactive protein (CRP), and bronchial washing results (i.e., AFB smear, mycobacterial culture, and Gram stain culture). Bronchoscopic findings were classified into seven subtypes: actively caseating, edematous-hyperemic, fibrostenotic, tumorous, granular, ulcerative, and nonspecific bronchitic [4]. Cases with two or more endobronchial involvement subtypes were classified by the dominant form. The involved site of EBTB was classified as the trachea, main bronchus, right bronchus intermedius (RBI), or lobar bronchus. EBTB with two or more involved bronchial levels was defined as multiple-level involvement, which was described separately for each lesion.

### Ethics approval

This study was approved by the Institutional Review Board for Human Research of Yonsei University Wonju Severance Christian Hospital (CR-319113). The requirement for informed consent was waived because of the retrospective nature of the study. Further, patient data were deidentified before data access and analysis.

### Statistical analysis

Data are presented as the median with interquartile range (IQR) or the number of patients with the percentages of the total. Continuous variables were compared using the Mann-Whitney U test. Categorical variables were compared using the Pearson’s chi-square test or the Fisher’s exact test. A *p*-value less than 0.05 was considered statistically significant. Statistical analysis was conducted using SPSS for Windows, version 23.0 (IBM Co., Chicago, IL, USA).

## Results

### Baseline characteristics

A total of 216 patients were included and the baseline characteristics are shown in Table 1. The median age of patients was 73 years, 142 (65.7%) were female, and the median BMI was 21.4 kg/m^2^. The number of patients that never smoked was 155 (71.8%) and those with a history of previous MTB treatment totaled 32 (14.8%) patients. The common comorbidities were diabetes (n = 47, 21.8%) and chronic kidney disease (n = 9, 4.2%). The most common predominant symptoms included cough or sputum (n = 132, 61.1%) followed by hemoptysis (n = 12, 5.6%); however, 61 (28.2%) patients were asymptomatic at the time of diagnosis. In chest CT findings, pulmonary consolidation and cavities were observed in 128 (59.3%) and 31 (14.4%) patients, respectively. On the contrary, 32 (14.8%) patients had no active lung parenchymal lesions on images. The median WBC, ESR, and CRP were 6,820 /μL, 50 mm/h, and 1.76 mg/dL, respectively. In bronchial washing specimens, AFB smears were positive in 149 (69.0%) patients.

**Table 1:** Baseline characteristics.

	Overall (*n* = 216)
Age, years	73 (55–79)
Gender, female	142 (65.7%)
Body mass index, kg/m^2^	21.4 (19.7–23.6)
Smoking history	
Never smoker	155 (71.8%)
History of MTB treatment	32 (14.8%)
Comorbidities	
Diabetes	47 (21.8%)
Chronic kidney disease	9 (4.2%)
Respiratory symptoms*	
Cough or sputum	132 (61.1%)
Hemoptysis	12 (5.6%)
Asymptomatic	61 (28.2%)
Chest computed tomographic findings*	
Consolidation	128 (59.3%)
Cavity	31 (14.4%)
No active lung parenchymal lesion	32 (14.8%)
Laboratory findings	
White blood cell, /μL	6,820 (5,565–8,625)
Erythrocyte sedimentation rate, mm/h	50 (29–67)
C-reactive protein, mg/dL	1.76 (0.50–5.29)
AFB stain^†^	
0	67 (31.0%)
1+	68 (31.5%)
2+	39 (18.1%)
3+	29 (13.4%)
4+	13 (6.0%)

* Cases are duplicated.

^†^ Positive acid-fast bacilli (AFB) stains were described as 1+ to 4+ (1+, 1–9 AFB/100 fields; 2+, 1–9 AFB/10 fields; 3+, 1–9 AFB/field; and 4+, > 9 AFB/field).

Values are presented as median (interquartile range) or count (percentage).

MTB, *Mycobacterium tuberculosis*; AFB, acid-fast bacilli

### Bronchoscopic findings

The bronchoscopic features of EBTB are shown in Table 2. Actively caseating, edematous-hyperemic, fibrostenotic, and nonspecific bronchitic type were observed in 114 (52.8%), 32 (14.8%), 33 (15.3%), and 18 (8.3%) patients, respectively. The most commonly involved site was the lobar bronchi (n = 194, 89.8%) followed by the main bronchi or the right bronchus intermedius (n = 42, 19.4%), and trachea (n = 11, 5.1%), respectively. Twenty-nine (13.4%) patients had multiple levels of bronchial involvement.

**Table 2:** Bronchoscopic features.

	Overall (*n* = 216)
Bronchoscopic finding	
Actively caseating	114 (52.8%)
Edematous-hyperemic	32 (14.8%)
Fibrostenotic	33 (15.3%)
Other types*	37 (17.1%)
Site involved^‡^	
Trachea	11 (5.1%)
Main bronchi or RBI	42 (19.4%)
Lobar bronchi	194 (89.8%)
Levels involved	
Single	187 (86.6%)
Multiple	29 (13.4%)

* Including tumorous (n = 5), granular (n = 5), ulcerative (n = 9), and nonspecific bronchitic type (n = 18)

^‡^ EBTB with multiple-level involvement was counted for each involvement.

Values are presented as count (percentage).

RBI, right bronchus intermedius; EBTB, endobronchial tuberculosis

### Microbiological characteristics

Microbiological characteristics of the bronchial washing samples are shown in Table 3. Apart from MTB, bacteria were detected in 42 (19.4%) patients and 6 (2.8%) of them had multiple microbial species. A total of 19 bacterial species were isolated. Apart from MTB, the most common microorganisms identified were *Staphylococcus aureus* (n = 14, 33.3%) including 8 cases of methicillin-sensitive *S*. *aureus* (MSSA) and 6 cases of methicillin-resistant *S*. *aureus* (MRSA). The second most common were *Klebsiella* species (n = 12, 28.6%) including 10 cases of *Klebsiella pneumonia* and 2 cases of *Klebsiella oxytoca*, followed by *Streptococcus* species (n = 5, 11.9%), *Enterobacter* species (n = 4, 9.5%), *Pseudomonas aeruginosa* (n = 3, 7.1%), and *Acinetobacter baumannii* (n = 2, 4.8%). Comparison of the clinical characteristics of EBTB patients with or without microorganisms isolated in bronchial washing is shown in Table 4. Consolidation in chest CT findings tended to be more frequent in patients with microorganisms isolated (71.4%) than those without microorganisms isolated in bronchial washing (56.3%; *P* = 0.082). There were no differences in baseline characteristics, symptoms, or laboratory and bronchoscopic findings. Additionally, comparison of the clinical characteristics of EBTB patients with or without *S*. *aureus* isolated in bronchial washing is presented in S1 Table of the S1 Appendix. There were no differences in baseline characteristics, symptoms, or laboratory and CT or bronchoscopic findings either.

**Table 3:** Microbiological characteristics of bronchial washing fluid.

	Overall (*n* = 216)
Patients with detected microorganisms	42 (19.4%)
Single microorganisms	36 (16.7%)
Poly-microorganisms	6 (2.8%)
Total number of isolated microorganisms	19
Identification of microorganisms	
*Staphylococcus aureus*	14 (33.3%)
MSSA	8
MRSA	6
*Klebsiella* species	12 (28.6%)
*Klebsiella pneumoniae*	10
*Klebsiella oxytoca*	2
*Streptococcus* species	5 (11.9%)
*Streptococcus agalactiae*	1
*Streptococcus anginosus*	1
*Streptococcus constellatus*	1
*Streptococcus haemolyticus*	1
*Streptococcus pneumoniae*	1
*Enterobacter* species	4 (9.5%)
*Enterobacter cloacea*	3
*Enterobacter aerogenes*	1
*Pseudomonas aeruginosa*	3 (7.1%)
*Acinetobacter baumannii*	2 (4.8%)
*Neisseria* species	2 (4.8%)
Miscellaneous	6 (14.3%)
*Aeromonas hydophilia*	1
*Citrobacter koseri*	1
*Enterococcus* species	1
*Escherichia coli*	1
*Proteus mirabilis*	1
*Stenotrophomonas maltophilia*	1

Values are presented as count (percentage).

MRSA, methicillin-resistant *Staphylococcus aureus*; MSSA, methicillin-sensitive *Staphylococcus aureus*

**Table 4:** Comparison of clinical characteristics with or without microorganisms in bronchial washing fluid.

	Microorganisms		*p*-value
	Yes (*n* = 42)	No (*n* = 174)	
Age, years	74 (61–78)	72 (54–79)	0.362
Gender, female	25 (59.5%)	117 (67.2%)	0.368
Body mass index, kg/m^2^	21.2 (19.3–23.6)	21.4 (19.9–23.7)	0.716
Ex or current smoker	14 (33.3%)	47 (27.0%)	0.447
Comorbidities			
History of MTB treatment	9 (21.4%)	23 (13.2%)	0.224
Diabetes	8 (19.0%)	39 (22.4%)	0.835
Chronic kidney disease	1 (2.4%)	8 (4.6%)	> 0.999
Respiratory symptoms*			
Cough or sputum	22 (52.4%)	110 (63.2%)	0.219
Hemoptysis	2 (4.8%)	10 (5.7%)	> 0.999
Asymptomatic	12 (28.6%)	49 (28.2%)	> 0.999
Chest CT findings*			
Consolidation	30 (71.4%)	98 (56.3%)	0.082
Cavity	8 (19.0%)	23 (13.2%)	0.333
Laboratory findings			
White blood cell, /μL	6,680 (5,470–8,773)	6,860 (5,565–8,585)	0.804
Erythrocyte sedimentation rate, mm/h	47 (29–64)	51 (29–68)	0.416
C-reactive protein, mg/dL	1.40 (0.45–3.58)	1.93 (0.50–5.56)	0.382
Bronchoscopic finding			
Actively caseating	17 (40.5%)	97 (55.7%)	0.086
Edematous-hyperemic	8 (19.0%)	24 (13.8%)	0.467
Fibrostenotic	9 (21.4%)	24 (13.8%)	0.235
Site involved^‡^			
Trachea	2 (4.8%)	9 (5.2%)	> 0.999
Main bronchi or RBI	5 (11.9%)	37 (21.3%)	0.198
Lobar bronchi	38 (90.5%)	156 (89.7%)	> 0.999
Multiple levels involved	3 (7.1%)	26 (14.9%)	0.217

* Cases are duplicated.

^‡^ EBTB with multiple-level involvement was counted for each involvement.

Values are presented as median (interquartile range) or count (percentage).

MTB, *Mycobacterium tuberculosis*; CT, computed tomography; RBI, right bronchus intermedius

## Discussion

This study investigated the composition of bacteria in the lower respiratory tract at patients with EBTB. We demonstrated that 19.4% of EBTB patients had microorganisms in bronchial washing specimens. With the exception of MTB, the most common bacteria included *S*. *aureus* followed by *Klebsiella*, *Streptococcus*, and *Enterobacter* species.

The lungs have traditionally been considered sterile, but a growing number of studies have shown that the lung and the respiratory tract contain a diverse community of microorganisms [19]. The constitution of intrapulmonary microorganisms is affected by the dynamics of microorganisms immigration and elimination in the respiratory tract [20]. Furthermore, airway epithelial cells play a central role in the dynamics of microorganisms by various mechanisms, including structural barrier function, mucociliary clearance, and the production of antimicrobial peptides (AMPs), reactive oxygen (ROS), and a range of cytokines, chemokines and growth factors [21, 22].

Chronic lung disease induces persistent airway epithelial cell damage, which can affect regional growth conditions and the respiratory microbial community, and changes in lung microorganisms contribute to the progression of underlying lung diseases, establishing a vicious cycle of disease [23]. Previous studies have shown that some structural lung diseases have specific patterns of composition in the respiratory bacterial community that can be associated with the pathogenesis and prognosis of disease [8, 10, 24]. The most frequently isolated bacteria in COPD patients are *Haemophilus influenzae* and *Pseudomonas aeruginosa*, and *P*. *aeruginosa* colonization is also reported to be associated with exacerbations [25]. Recent studies have found a relative abundance of *Staphylococcus* and *Streptococcus* species in patients with idiopathic pulmonary fibrosis and have suggested its possible association with the pathogenesis of disease [12, 26]. In bronchiectasis, the role of respiratory microorganisms has been largely investigated in the same manner [27]. Frequently isolated and potentially pathogenic microorganisms include *H*. *influenza*, *P*. *aeruginosa*, and *Streptococcus pneumonia*. *P*. *aeruginosa* is associated with impaired lung function and increased mortality in bronchiectasis [10, 28].

In pulmonary tuberculosis, several studies have described the characteristics of the lung microorganisms as well [29, 30]. The respiratory bacterial composition in patients with pulmonary tuberculosis showed more diversity than those of healthy controls in its composition and many unique bacteria such as *Stenotrophomonas*, *Cupriavidus*, *Caulobacter*, *Pseudomonas*, *Thermus*, and *Sphingomonas* were found [30]. Additionally, respiratory bacterial pathogens were detected in sputum at 29% patients, and the common organisms were *H*. *influenzae* (21%) and *S*. *pneumonia* (8%) [29]. Some studies have reported a possible association between the presence of respiratory microorganisms and poorer prognosis in pulmonary tuberculosis [29, 31].

However, respiratory microorganisms in patients with EBTB have not been investigated in the literature. The reported prevalence of bacterial co-infection in patients with pulmonary tuberculosis varies greatly (from 17% to 44%) [29, 31, 32]. In this study, the prevalence of bacterial co-infection in patients with EBTB was 19%. In addition, the most common respiratory microorganism in EBTB was *S*. *aureus* isolated in 33.3% of the patients with detected microorganisms, followed by *Klebsiella* and *Streptococcus* species. These findings show that the microorganisms in EBTB are distinct from the other respiratory diseases discussed above. *H*. *influenza* and *P*. *aeruginosa*, which are the most common microorganisms in COPD and bronchiectasis, were isolated in 0 and 3 (7.1%) of patients in our study.

Interestingly, *S*. *aureus*, the most common bacterial isolate in this study, is a well-established predominant potential pathogen in patients with cystic fibrosis [33]. Further, this microorganism is frequently isolated in patients with MTB based on previous studies [31, 32]. *S*. *aureus* attaches primarily to the mucus component of the airway epithelium and can evade the host immune response and cause persistent colonization by biofilm formation [34]. Further studies are needed to establish the predominance of *S*. *aureus* in EBTB and its clinical significance.

This study has some limitations. First, this is a retrospective study so we could not investigate the causal relationship between microorganisms and EBTB. Second, there was no statistical significance for clinical differences between patients with or without bacteria isolated in the bronchial washing. Since many of the patients diagnosed with EBTB in this tertiary referral hospital had been later transferred to regional centers for follow-up care, there were some limitations on data collection for the treatment outcomes such as the presence of bronchial stenosis after treatment completed or changes in pulmonary functions of these patients. Further investigations are needed on the clinical significance of these organisms. Third, bacterial identification in bronchial washing was done according to conventional culture methods, so microorganisms information on non-culturable bacteria could not be determined. Finally, we did not exclude patients who received antibiotics prior to the study. However, most patients with EBTB are referred to a tertiary referral hospital without improvement during antibiotic treatment, which may reflect the real-world clinical setting.

## Conclusions

A variety of microorganisms were isolated in the lower respiratory tract at patients with EBTB and changes in the composition of microorganisms take place in the airways of those with EBTB. Further studies are needed to investigate the clinical significance of this finding.

## Supporting information

S1 Appendix (DOCX)

S1 Data (XLSX)

## Abbreviations

AFB: acid-fast bacilli
BMI: body mass index
COPD: chronic obstructive pulmonary disease
CRP: C-reactive protein
CT: computed tomography
EBTB: endobronchial tuberculosis
ESR: erythrocyte sedimentation rate
MTB: *Mycobacterium tuberculosis*
RBI: right bronchus intermedius

## References

[1] Shim YS. Endobronchial tuberculosis. Respirology. 1996;1: 95–106. 10.1111/j.1440-1843.1996.tb00017.x . 9434324

[2] Lee JH, Park SS, Lee DH, Shin DH, Yang SC, Yoo BM. Endobronchial tuberculosis. Clinical and bronchoscopic features in 121 cases. Chest. 1992;102: 990–994. 10.1378/chest.102.4.990 . 1395814

[3] Jung SS, Park HS, Kim JO, Kim SY. Incidence and clinical predictors of endobronchial tuberculosis in patients with pulmonary tuberculosis. Respirology. 2015;20: 488–495. 10.1111/resp.12474 . 25620110 PMC4409824

[4] Chung HS, Lee JH. Bronchoscopic assessment of the evolution of endobronchial tuberculosis. Chest. 2000;117: 385–392. 10.1378/chest.117.2.385 . 10669679

[5] Ozkaya S, Bilgin S, Findik S, Kok HC, Yuksel C, Atici AG. Endobronchial tuberculosis: histopathological subsets and microbiological results. Multidiscip Respir Med. 2012;7: 34 10.1186/2049-6958-7-34 . 23088170 PMC3488328

[6] Um SW, Yoon YS, Lee SM, Yim JJ, Yoo CG, Chung HS, et al Predictors of persistent airway stenosis in patients with endobronchial tuberculosis. Int J Tuberc Lung Dis. 2008;12: 57–62. . 18173878

[7] Cabello H, Torres A, Celis R, El-Ebiary M, Puig de la Bellacasa J, Xaubet A, et al Bacterial colonization of distal airways in healthy subjects and chronic lung disease: a bronchoscopic study. Eur Respir J. 1997;10: 1137–1144. 10.1183/09031936.97.10051137 . 9163659

[8] Wang JX, Li HQ, Zhang F, Ning W. Systemic inflammation and the effects of short-term antibiotic treatment for PPM positive patients with stable COPD. Int J Chron Obstruct Pulmon Dis. 2019;14: 1923–1932. 10.2147/copd.s217971 . 31692553 PMC6711567

[9] Hugle T. Immunology of fibrotic lung disease: managing infections whilst preventing autoimmunity? J Inflamm Res. 2011;4: 21–27. 10.2147/jir.s10602 . 22096366 PMC3218744

[10] Loebinger MR, Wells AU, Hansell DM, Chinyanganya N, Devaraj A, Meister M, et al Mortality in bronchiectasis: a long-term study assessing the factors influencing survival. Eur Respir J. 2009;34: 843–849. 10.1183/09031936.00003709 . 19357155

[11] Zalacain R, Sobradillo V, Amilibia J, Barron J, Achotegui V, Pijoan JI, et al Predisposing factors to bacterial colonization in chronic obstructive pulmonary disease. Eur Respir J. 1999;13: 343–348. 10.1034/j.1399-3003.1999.13b21.x . 10065679

[12] Molyneaux PL, Maher TM. The role of infection in the pathogenesis of idiopathic pulmonary fibrosis. Eur Respir Rev. 2013;22: 376–381. 10.1183/09059180.00000713 . 23997064 PMC9487348

[13] Aksamit TR, O’Donnell AE, Barker A, Olivier KN, Winthrop KL, Daniels MLA, et al Adult Patients With Bronchiectasis: A First Look at the US Bronchiectasis Research Registry. Chest. 2017;151: 982–992. 10.1016/j.chest.2016.10.055 . 27889361 PMC6026266

[14] Esteban J, Garcia-Coca M. Mycobacterium Biofilms. Front Microbiol. 2017;8: 2651 10.3389/fmicb.2017.02651 . 29403446 PMC5778855

[15] Mandell LA, Wunderink RG, Anzueto A, Bartlett JG, Campbell GD, Dean NC, et al Infectious Diseases Society of America/American Thoracic Society consensus guidelines on the management of community-acquired pneumonia in adults. Clin Infect Dis. 2007;44 Suppl 2: S27–72. 10.1086/511159 . 17278083 PMC7107997

[16] Lee N, Kim SH, Kwon W, Lee MK, Yong SJ, Shin KC, et al The effects of bronchoscope diameter on the diagnostic yield of transbronchial lung biopsy of peripheral pulmonary nodules. Tuberc Respir Dis (Seoul). 2014;77: 251–257. 10.4046/trd.2014.77.6.251 . 25580141 PMC4286782

[17] Meyer KC, Raghu G, Baughman RP, Brown KK, Costabel U, du Bois RM, et al An official American Thoracic Society clinical practice guideline: the clinical utility of bronchoalveolar lavage cellular analysis in interstitial lung disease. Am J Respir Crit Care Med. 2012;185: 1004–1014. 10.1164/rccm.201202-0320ST . 22550210

[18] Diagnostic Standards and Classification of Tuberculosis in Adults and Children. This official statement of the American Thoracic Society and the Centers for Disease Control and Prevention was adopted by the ATS Board of Directors, July 1999. This statement was endorsed by the Council of the Infectious Disease Society of America, September 1999. Am J Respir Crit Care Med. 2000;161: 1376–1395. 10.1164/ajrccm.161.4.16141 . 10764337

[19] Dickson RP, Erb-Downward JR, Martinez FJ, Huffnagle GB. The Microbiome and the Respiratory Tract. Annu Rev Physiol. 2016;78: 481–504. 10.1146/annurev-physiol-021115-105238 . 26527186 PMC4751994

[20] Dickson RP, Erb-Downward JR, Freeman CM, McCloskey L, Beck JM, Huffnagle GB, et al Spatial Variation in the Healthy Human Lung Microbiome and the Adapted Island Model of Lung Biogeography. Ann Am Thorac Soc. 2015;12: 821–830. 10.1513/AnnalsATS.201501-029OC . 25803243 PMC4590020

[21] Bals R, Hiemstra PS. Innate immunity in the lung: how epithelial cells fight against respiratory pathogens. Eur Respir J. 2004;23: 327–333. 10.1183/09031936.03.00098803 . 14979512

[22] Hiemstra PS, McCray PB Jr., Bals R. The innate immune function of airway epithelial cells in inflammatory lung disease. Eur Respir J. 2015;45: 1150–1162. 10.1183/09031936.00141514 . 25700381 PMC4719567

[23] Dickson RP, Martinez FJ, Huffnagle GB. The role of the microbiome in exacerbations of chronic lung diseases. Lancet. 2014;384: 691–702. 10.1016/s0140-6736(14)61136-3 . 25152271 PMC4166502

[24] Molyneaux PL, Cox MJ, Willis-Owen SA, Mallia P, Russell KE, Russell AM, et al The role of bacteria in the pathogenesis and progression of idiopathic pulmonary fibrosis. Am J Respir Crit Care Med. 2014;190: 906–913. 10.1164/rccm.201403-0541OC 25184687 PMC4299577

[25] Rosell A, Monso E, Soler N, Torres F, Angrill J, Riise G, et al Microbiologic determinants of exacerbation in chronic obstructive pulmonary disease. Arch Intern Med. 2005;165: 891–897. 10.1001/archinte.165.8.891 15851640

[26] Han MK, Zhou Y, Murray S, Tayob N, Noth I, Lama VN, et al Lung microbiome and disease progression in idiopathic pulmonary fibrosis: an analysis of the COMET study. Lancet Respir Med. 2014;2: 548–556. 10.1016/s2213-2600(14)70069-4 . 24767767 PMC4142525

[27] Chalmers JD, Hill AT. Mechanisms of immune dysfunction and bacterial persistence in non-cystic fibrosis bronchiectasis. Mol Immunol. 2013;55: 27–34. 10.1016/j.molimm.2012.09.011 . 23088941

[28] Tunney MM, Einarsson GG, Wei L, Drain M, Klem ER, Cardwell C, et al Lung microbiota and bacterial abundance in patients with bronchiectasis when clinically stable and during exacerbation. Am J Respir Crit Care Med. 2013;187: 1118–1126. 10.1164/rccm.201210-1937OC . 23348972 PMC3734618

[29] Shimazaki T, Taniguchi T, Saludar NRD, Gustilo LM, Kato T, Furumoto A, et al Bacterial co-infection and early mortality among pulmonary tuberculosis patients in Manila, The Philippines. Int J Tuberc Lung Dis. 2018;22: 65–72. 10.5588/ijtld.17.0389 . 29297428

[30] Cui Z, Zhou Y, Li H, Zhang Y, Zhang S, Tang S, et al Complex sputum microbial composition in patients with pulmonary tuberculosis. BMC Microbiol. 2012;12: 276 10.1186/1471-2180-12-276 . 23176186 PMC3541192

[31] Shirai M, Hayakawa H, Uchiyama H, Chida K, Nakamura H. Clinical significance of potential pathogenic microorganisms of sputum in patients with pulmonary tuberculosis. Respirology. 2001;6: 311–315. 10.1046/j.1440-1843.2001.00349.x . 11844122

[32] Ishikawa S, Igari H, Yamagishi K, Takayanagi S, Yamagishi F. Microorganisms isolated at admission and treatment outcome in sputum smear-positive pulmonary tuberculosis. J Infect Chemother. 2019;25: 45–49. 10.1016/j.jiac.2018.10.005 . 30414723

[33] Salsgiver EL, Fink AK, Knapp EA, LiPuma JJ, Olivier KN, Marshall BC, et al Changing Epidemiology of the Respiratory Bacteriology of Patients With Cystic Fibrosis. Chest. 2016;149: 390–400. 10.1378/chest.15-0676 . 26203598 PMC5831653

[34] Paharik AE, Horswill AR. The Staphylococcal Biofilm: Adhesins, Regulation, and Host Response. Microbiol Spectr. 2016;4: 10.1128/microbiolspec.VMBF-0022-2015 . 27227309 PMC4887152

