# Supplementary material for: A variety of bacterial aetiologies in the lower respiratory tract at patients with endobronchial tuberculosis
Source: PLoS One. 2020 Jun 25;15(6):e0234558. doi: 10.1371/journal.pone.0234558 (PMC7316277; doi:10.1371/journal.pone.0234558)
Supplement: S1 Appendix — (DOCX) [file pone.0234558.s001.docx]

**Supplementary Appendix**

**A variety of bacterial aetiologies in the lower respiratory tract at patients with endobronchial tuberculosis**

Sae Byol Kim^1^ ^¶^, Won-Yeon Lee ^1^ ^¶^, Ji-Ho Lee^1^, Seok Jeong Lee^1^, Myoung Kyu Lee^1^, Sang-Ha Kim^1^, Young Uh^2^, Soon-Hee Jung^3^, Beomsu Shin^1*^

^1^Department of Internal Medicine, Yonsei University Wonju College of Medicine, Wonju, Republic of Korea

^2^Department of Laboratory Medicine, Yonsei University Wonju College of Medicine, Wonju, Republic of Korea

^3^Department of Pathology, Yonsei University Wonju College of Medicine, Wonju, Republic of Korea

*Corresponding author

E-mail: [bsshin83@gmail.com](mailto:bsshin83@gmail.com) (BS)

^¶^ These authors contributed equally to this work.

**Table S1. Comparison of clinical characteristics with or without *Staphylococcus aureus* in bronchial washing fluid.**

|  | *Staphylococcus aureus* | | *p*-value |
| --- | --- | --- | --- |
|  | Yes (*n* = 14) | No (*n* = 202) |  |
| Age, years | 74 (47 – 83) | 73 (56 – 79) | 0.677 |
| Gender, female | 12 (85.7%) | 130 (64.4%) | 0.146 |
| Body mass index, kg/m^2^ | 21.3 (18.7 – 23.9) | 21.4 (19.9 – 23.6) | 0.873 |
| Ex or current smoker | 3 (21.4%) | 58 (28.7%) | 0.762 |
| Comorbidities |  |  |  |
| History of MTB treatment | 3 (21.4%) | 29 (14.4%) | 0.442 |
| Diabetes | 1 (7.1%) | 46 (22.8%) | 0.312 |
| Chronic kidney disease | 0 | 9 (4.5%) | > 0.999 |
| Respiratory symptoms^*^ |  |  |  |
| Cough or sputum | 7 (50.0%) | 125 (61.9%) | 0.406 |
| Hemoptysis | 0 | 12 (5.9%) | > 0.999 |
| Asymptomatic | 7 (50.0%) | 54 (26.7%) | 0.072 |
| Chest CT findings^*^ |  |  |  |
| Consolidation | 8 (57.1%) | 120 (59.4%) | > 0.999 |
| Cavity | 2 (14.3%) | 29 (14.4%) | > 0.999 |
| Laboratory findings |  |  |  |
| White blood cell, /µL | 6,450 (5,595 – 7,053) | 6,920 (5,555 – 8,733) | 0.267 |
| Erythrocyte sedimentation rate, mm/h | 30 (15 – 60) | 51 (30 – 67) | 0.138 |
| C-reactive protein, mg/dL | 1.40 (0.40 – 3.20) | 1.80 (0.50 – 5.33) | 0.615 |
| Bronchoscopic finding |  |  |  |
| Actively caseating | 5 (35.7%) | 109 (54.0%) | 0.269 |
| Edematous-hyperemic | 2 (14.3%) | 30 (14.9%) | > 0.999 |
| Fibrostenotic | 4 (28.6%) | 29 (14.4%) | 0.238 |
| Site involved^‡^ |  |  |  |
| Trachea | 1 (7.1%) | 10 (5.0%) | 0.530 |
| Main bronchi or RBI | 2 (14.3%) | 40 (19.8%) | > 0.999 |
| Lobar bronchi | 13 (92.9%) | 181 (89.6%) | > 0.999 |
| Multiple levels involved | 2 (14.3%) | 27 (13.4%) | > 0.999 |

^*^ Cases are duplicated.

^‡^ EBTB with multiple-level involvement was counted for each involvement.

Values are presented as median (interquartile range) or count (percentage).

MTB, *Mycobacterium* *tuberculosis*; CT, computed tomography; RBI, right bronchus intermedius
